# Triptolide inhibits ovarian cancer growth and metastasis via reprogramming of tumor-associated macrophages

**DOI:** 10.15698/cst2026.07.319

**Published:** 2026-07-07

**Authors:** Ziqi Chen, Jie Zhang, Jianting Lao, Chaoqin Yu, Hong Yang

**Affiliations:** 1 Institute of Oncology, Municipal Hospital of Traditional Chinese Medicine, Shanghai University of Traditional Chinese Medicine, Shanghai, China; 2 Science and Technology Innovation Center, Shanghai Municipal Hospital of Traditional Chinese Medicine, Shanghai University of Traditional Chinese Medicine, Shanghai, China; 3 Central Laboratory for Science and Technology, Longhua Hospital, Shanghai University of Traditional Chinese Medicine, Shanghai, 200032, China; 4 Department of Gynecology, Shanghai Municipal Hospital of Traditional Chinese Medicine, Shanghai University of Traditional Chinese Medicine, Shanghai, 200071, China; 5 Department of Traditional Chinese Gynecology, The First Affiliated Hospital of Naval Medical University, Shanghai, 200433, China

**Keywords:** Triptolide, ovarian cancer, macrophage reprograming, Nrf2, tumor-associated macrophages (TAMs)

## Abstract

Triptolide, an extract from the Chinese herb Thunder God Vine, a compound renowned for its anti-cancer properties, exhibits an elusive mechanism of action. While extensive research has elucidated its direct effects on cancer cells, the indirect impact on non-tumor cells within the cancer microenvironment remains poorly understood. In this study, we investigated the influence of Triptolide on tumor-associated macrophages (TAMs), pivotal contributors to ovarian cancer progression. Using cell culture and promoter assay, cellular viability assessment, cell clock assay, transwell assays, flow cytometry, ELISA, TUNEL staining, and mouse models, we found that Triptolide does not significantly affect macrophage proliferation or survival; instead, it induces differentiation of naive macrophages towards the M1 phenotype and reprograms M2-polarized macrophages into a similar inflammatory state. These observations suggest that modulation of TAMs may partially underlie Triptolide’s hindrance of ovarian cancer progression. Mechanistically, we reveal that Triptolide inhibits Nrf2 transcription - a master regulator governing anti-inflammatory responses in macrophages. Functional gain- and loss-of-function studies further confirmed that Nrf2 inhibition is essential for Triptolide-mediated TAM reprogramming and subsequent suppression of cancer cell progression. Co-culturing with macrophages substantially enhances ovarian cancer cell growth, invasion, and migration; however, all these effects are abrogated by treatment with Triptolide. Collectively, our findings indicate that the suppression of ovarian cancer by Triptolide is mediated in part through its capacity to reprogram TAMs via the Nrf2 pathway.

## INTRODUCTION

Ovarian cancer presents a diverse range of clinical characteristics due to its origin in the cells of the ovaries 1. Often asymptomatic in early stages, it may eventually manifest through non-specific symptoms such as abdominal bloating, pain, difficulty eating, or quick satiety 1. As the disease progresses, more pronounced symptoms can occur, including pelvic discomfort, changes in bowel habits, frequent urination, and general abdominal distension 2. Ovarian cancer’s insidious nature often leads to late-stage diagnosis, where the prognosis is poorer 2. Unlike other gynecological malignancies, specific early detection tests for ovarian cancer are not widely available, making regular check-ups and attention to bodily changes critical for early detection 2. Advanced stages may be characterized by ascites, pleural effusion, or metastasis to distant organs 2. Due to the lack of specific symptoms and screening tests, vigilance for the subtle signs of ovarian cancer is paramount 3.

Triptolide, an extract from the Chinese herb Thunder God Vine, has garnered interest due to its potent anti-cancer properties across various cancer types 4–6. It has demonstrated effectiveness in inducing cell death, inhibiting cell proliferation, and reducing tumor growth 7. In multiple studies, triptolide has been shown to impede the progression of cancers such as pancreatic, colorectal, and breast cancer by triggering apoptosis and cell cycle arrest 7. Additionally, triptolide interferes with cancer cell metastasis and angiogenesis, crucial processes for cancer spread and nutrient acquisition 7. It’s also known to sensitize resistant cancer cells to chemotherapy and reduce inflammation, which is often conducive to cancer progression 4. However, while its direct effects on cancer cells have been extensively studied, its indirect effects on cancer through non-tumor cells in the cancer microenvironment has not been addressed 8. Moreover, due to its broad biological activity, further research is needed to fully understand its mechanisms and to develop safe, effective therapeutic strategies.

Previous studies have indicated that tumor-associated macrophages (TAMs) significantly influence the progression of ovarian cancer 9. TAMs, once recruited by the tumor environment, often adopt an M2 phenotype that promotes tumorigenesis, aiding in tissue remodeling, angiogenesis, and suppression of the immune response 10. As a result, a high density of TAMs within the tumor microenvironment has been associated with poor prognosis in ovarian cancer patients 11. Therapeutic strategies targeting the reprogramming of TAMs from a pro-tumorigenic M2 state to an anti-tumorigenic M1 state are considered promising for improving ovarian cancer outcomes 12. Here, we investigated how Triptolide influences TAMs, the key player in ovarian cancer cell growth and metastasis.

## RESULTS

### Triptolide inhibits ovarian cancer cell growth *in vitro*

**Figure 1:**
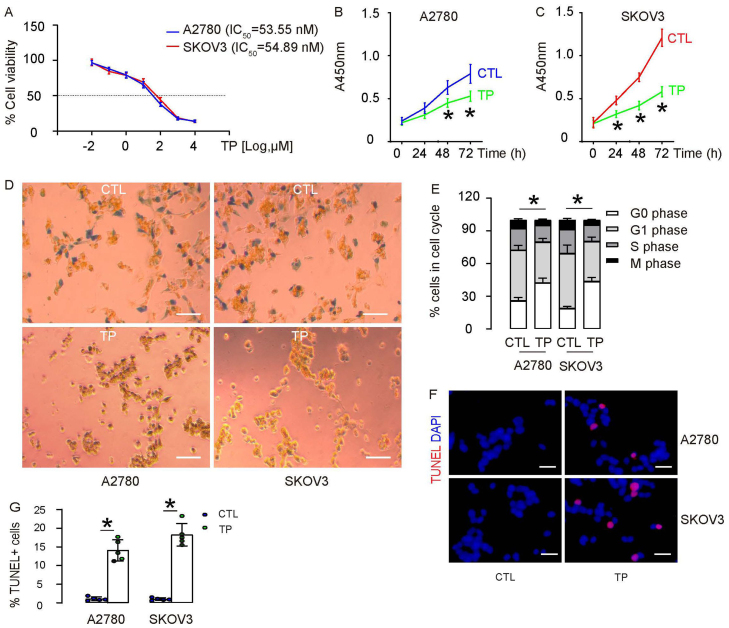
Triptolide inhibits ovarian cancer cell growth *in vitro*. **(A)** Determination of IC50 of Triptolide (TP) on A2780 and SKOV3 cells. **(B–C)** CCK-8 assay for A2780 **(B)** and SKOV3 cells **(C)**. **(D–E)** Cell clock assay to analyze A2780 and SKOV3 cells treated by 55nM Triptolide, shown by representative images **(D)**, and by quantification **(E)**. **(F)** A2780 and SKOV3 cells were treated by 55nM Triptolide. TUNEL staining was done to assess apoptosis, shown by representative images **(F)** and by quantification **(G)**. ^⁎^p < 0.05. N = 5. Scale bars are 50 μ m in panel D, and 20 μ m in panel F.

The effects of Triptolide on the growth of ovarian cancer cell lines A2780 and SKOV3 were assessed 13–15. The growth of ovarian cancer cells is attributed to two key effects: cell proliferation and cell apoptosis. Different doses of Triptolide were thus applied to A2780 and SKOV3 cells to allow determination of IC50 of Triptolide ( ∼ 55 nM), the dose required to inhibit cell growth by 50% (Figure 1**A**). Afterwords, an CCK-8 assay was performed, showing a decline in metabolic activity upon Triptolide treatment, suggesting cytotoxicity against ovarian cancer cells (Figure 1**B–C**). The Cell Clock Assay was then used to show that Triptolide treatment resulted in a significant reduction of cells in S phase and M phase, suggesting a potent inhibitory effect of Triptolide on cell cycle progression (Figure 1**D–E**). Next, TUNEL staining was used to quantify the percentage of apoptotic cells, showing that Triptolide significantly increased apoptotic ovarian cancer cells (Figure 1**F–G**).

### Triptolide reprograms macrophages towards a pro-inflammatory status

**Figure 2:**
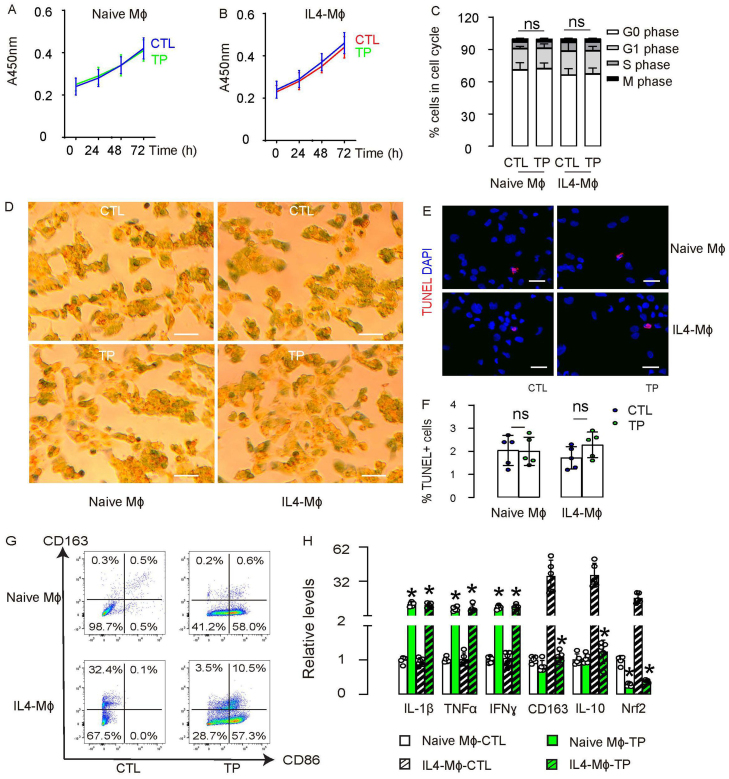
Triptolide reprograms macrophages towards a pro-inflammatory status. We used the concentration of Triptolide (TP; 55nM) that was found to induce IC50 of ovarian cells to treat naïve macrophages, or naïve macrophages that had been polarized to M2 by IL-4. **(A–B)** CCK-8 assay for naïve macrophages (naïve M ϕ ; A) and for IL-4-primed naïve macrophages (IL-4-M ϕ ; B). **(C–D)** Cell clock assay to analyze naïve M ϕ and IL-4-M ϕ treated by 55nM Triptolide, shown by quantification **(C)**, and by representative images **(D)**. **(E–F)** Naïve M ϕ and IL-4-M ϕ were treated by 55nM Triptolide. TUNEL staining was done to assess apoptosis, shown by representative images **(E)** and by quantification **(F)**. **(G)** Flow cytometry analysis of macrophage polarization by CD163 and CD86. **(H)** ELISA for IL-1 β , TNF α , IFN γ , CD163, IL-10 and Nrf2. ^⁎^p < 0.05. ns: non-significant. N = 5. Scale bars are 20 μ m.

We used the concentration of Triptolide (55 nM) that was found to induce IC50 of ovarian cells to treat naïve macrophages, or naïve macrophages that had been polarized to M2 by IL-4. Interestingly, this concentration of Triptolide didn’t significantly affect macrophage growth (Figure 2**A–B**) either through proliferation (Figure 2**C–D**) or through apoptosis (Figure 2**E–F**) but induced naive macrophages to differentiate into the M1 phenotype and reprogramed IL-4-polarized M2 macrophages into a similar proinflammatory state, by flow cytometry analysis of CD163+ M2 macrophages versus CD86+ M1 macrophages (Figure 2**G**) and by ELISA analysis of expression of proinflammatory cytokines IL-1 β , TNF α , IFN γ , and anti-inflammatory factors or cytokines CD163 and IL-10 (Figure 2**H**). This suggests that Triptolide may hinder ovarian cancer progression partially by modulating TAMs. Interestingly, Triptolide significantly inhibited expression of Nrf2 in macrophages (Figure 2**H**).

### Triptolide inhibits Nrf2 transcription to reprogram macrophages

**Figure 3:**
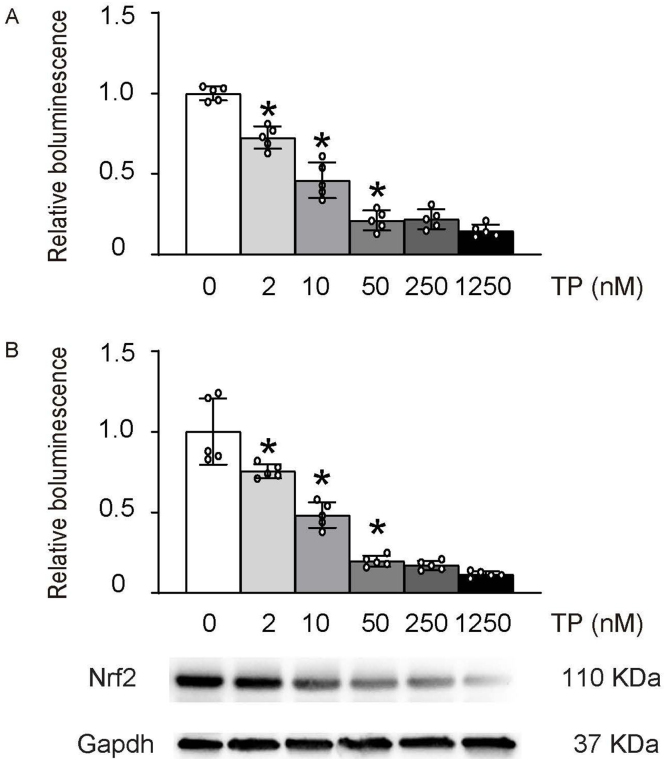
Triptolide inhibits Nrf2 transcription to reprogram macrophages. The potential effects of Triptolide (TP) on macrophage polarization via Nrf2 were examined. **(A)** Promoter assay for Nrf2 was performed using different concentration of TP. **(B)** Nrf2 levels by Western blot in macrophages treated with TP. ^⁎^p < 0.05, compared to the lower applied dose. N = 5.

Nuclear factor erythroid 2–related factor 2 (Nrf2) is pivotal in macrophage polarization, influencing their differentiation into pro-inflammatory M1 or anti-inflammatory M2 phenotypes. Recognized for orchestrating oxidative stress responses by regulating antioxidant protein expression, Nrf2’s activity is more commonly aligned with promoting the M2 phenotype. The delicate equilibrium between M1 and M2 macrophages within tumors is crucial for immune activities, with Nrf2 as a vital regulatory element. Given Triptolide’s documented impact on hepatic macrophages in a recent study 16, its potential effects on macrophage polarization via Nrf2 were examined. Our results indicated Triptolide directly and dose-dependently suppressed Nrf2 transcription, thereby modulating macrophage behavior, as confirmed by promoter assays (Figure 3**A**). We also examined Nrf2 protein expression data via Western blot, which showed a reduction in Nrf2 protein levels in sorted TAMs after triptolide treatment dose-dependently (Figure 3**B**). These data collectively indicate that triptolide suppresses Nrf2 activity at both transcriptional and post-translational levels. This suggests Triptolide’s mechanism may involve reprogramming macrophages by inhibiting Nrf2, the master regulator of anti-inflammatory responses.

### Triptolide reduces growth, invasion, and migration of ovarian cancer cells co-cultured with macrophages through Nrf2

**Figure 4:**
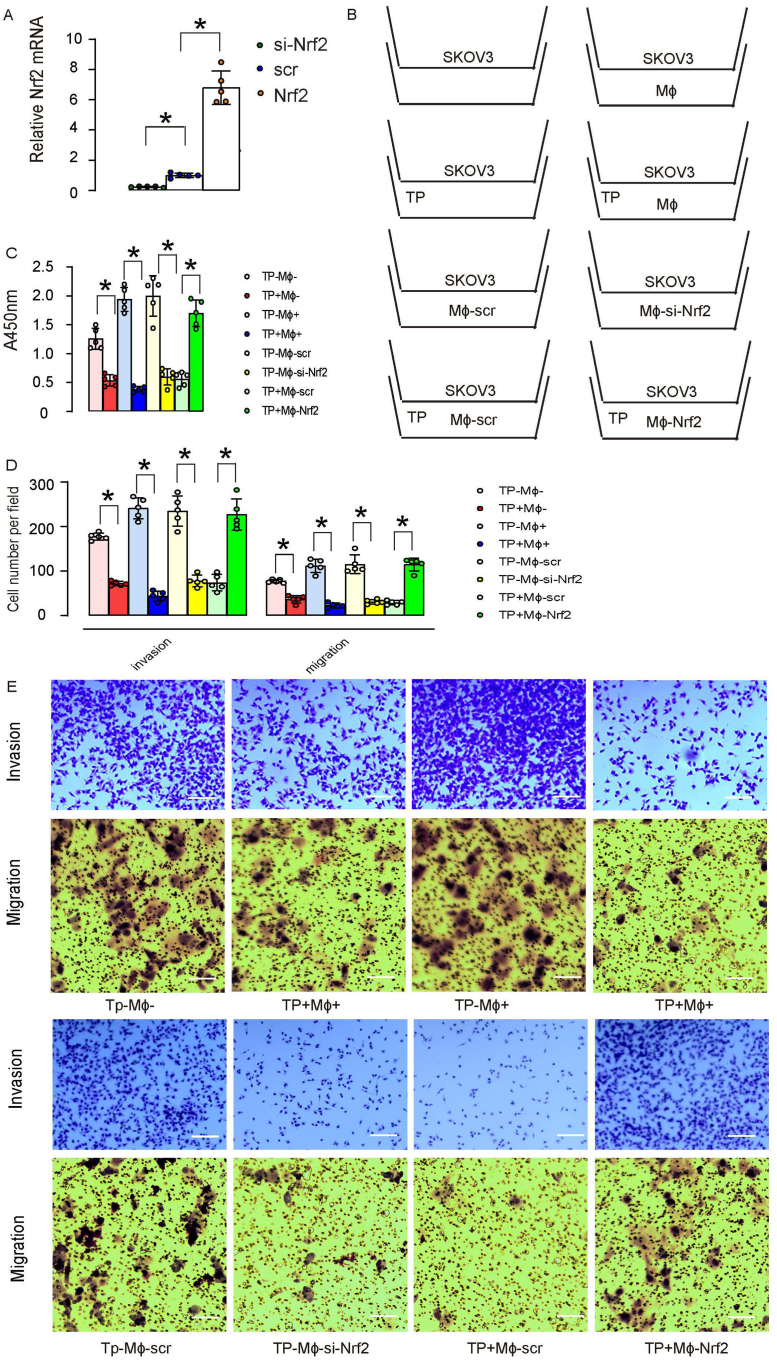
Triptolide reduces growth, invasion, and migration of ovarian cancer cells co-cultured with macrophages through Nrf2. **(A)** Validation of Nrf2 genetic modification in macrophages. Macrophages were transfected with either Nrf2-specific siRNA (**si-Nrf2**) for loss-of-function or an Nrf2 expression vector (**Nrf2**) for gain-of-function studies. Transfection with scramble (scr) was used as a control. RT-qPCR for Nrf2 was performed to confirm the effects. **(B)** Illustration of the experiment: Ovarian cancer cells SKOV3 were either cultured alone or in combination with macrophages (M ϕ ) or si-Nrf2-/scr-/Nrf2- transfected M ϕ , in the presence or absence of Triptolide (TP). **(C)** CCK-8 assay for SKOV3 cells. **(D–E)** Transwell invasion and migration assays shown by quantification **(D)** or by representative images **(E)**. ^⁎^p < 0.05. N = 5. Scale bars are 100 μ m.

To investigate whether Nrf2 is the functional target of TP in TAMs, we need to genetic loss-of-function and gain-of-function studies using a co-culture system with SKOV3 ovarian cancer cells. Firstly, we silenced Nrf2 in macrophages using specific siRNA (si-Nrf2) or overexpressed Nrf2 in macrophages (Nrf2) (Figure 4**A**).

To explore Triptolide’s impact on ovarian cancer progression, we conducted experiments where ovarian cancer cells were either cultured alone or in combination with macrophages, in the presence or absence of Triptolide (Figure 4**B**). Our findings showed that co-culture with macrophages significantly enhanced the growth of ovarian cancer cells, an effect mitigated by Triptolide treatment (Figure 4**C**). Additionally, our analysis on cell invasion and migration demonstrated that Triptolide effectively inhibited these processes (Figure 4**D–E**). Moreover, the effects of Triptolide on cancer cells appeared more pronounced in the presence of macrophages (Figure 4**C–E**). In examine whether Nrf2 is the functional target of TP in TAMs, we added several conditions: 1. Use of si-Nrf2-transfected macrophages to see whether it mimics the effects of TP; 2. Use of Nrf2-transfected macrophages in the presence of TP to see whether it suppresses the inhibitory effect of Triptolide (Figure 4**B**). We found that the knockdown of Nrf2 in macrophages significantly reduced the proliferation (Figure 4**C**), migration, and invasion (Figure 4**D–E**) of co-cultured ovarian cancer cells compared to the control (scramble; scr) group, mimicking the anti-tumor phenotype observed in the TP-treated group, suggesting that Nrf2 deficiency is sufficient to reprogram macrophages toward an anti-tumor state. Moreover, overexpression of Nrf2 in macrophages (Nrf2) significantly attenuated the effects of TP on cancer cells (Figure 4**C–E**). Collectively, these findings demonstrate that Triptolide exerts its anti-tumor effects by specifically targeting and inhibiting the Nrf2 pathway within the tumor-associated macrophage population.

### IL-4 mitigated triptolide’s anti-tumor efficacy *in vivo*

**Figure 5:**
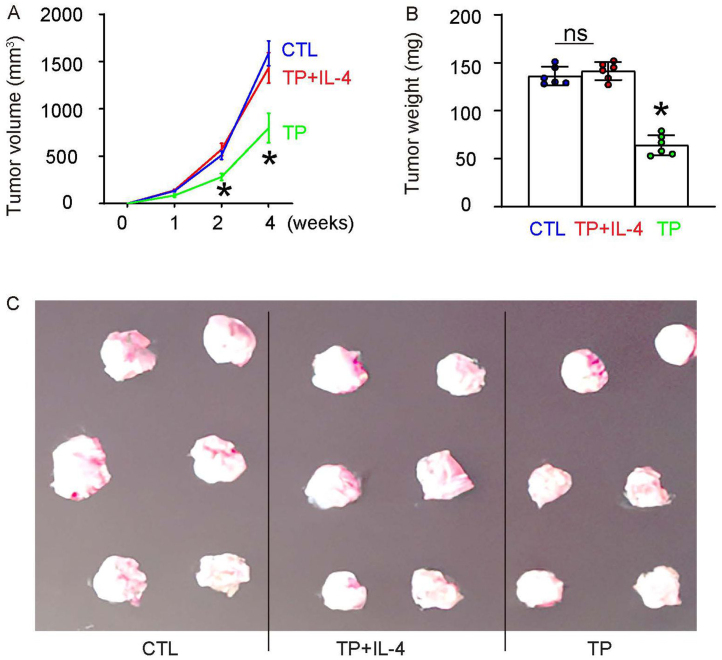
IL-4 mitigates triptolide’s anti-tumor efficacy *in vivo*. *In vivo* experiments using NOD/SCID mice with subcutaneous transplantation of SKOV3 cells was tested to prove the anti-tumor effects of Triptolide (TP; 55 μ g/kg ip injection, twice a week). Besides the control group that received saline instead of TP, another TP-treated group received the administration of IL-4 (TP+IL-4) to counter Triptolide’s influence on macrophage polarization. Tumor volume was followed for 4 weeks before the mice were sacrificed for analyzing tumor weight. **(A)** Tumor volume. **(B–C)** Tumor weight at 4 weeks by quantification **(B)** and by gross images **(C)**. ^⁎^p < 0.05. ns: non-significant. N = 6.

Finally, NOD/SCID mice were subcutaneously implanted with SKOV3 cells to assess Triptolide’s anti-cancer effects, contrasting them with and without IL-4 administration. IL-4 is a well-known M2 macrophage inducer and here we aimed to use it to counter Triptolide’s influence on macrophage polarization. We found that Triptolide significantly decreased the changes in tumor size (Figure 5**A**) and the tumor weight at sacrifice time (4 weeks after transplantation) (Figure 5**B–C**), effects that IL-4 partially reversed (Figure 5**A–C**), indicating Triptolide’s dual mechanism through direct ovarian cell impact and macrophage reprogramming.

### Significant anti-inflammatory TAMs are detected in triptolide-treated tumors that receive IL-4

**Figure 6:**
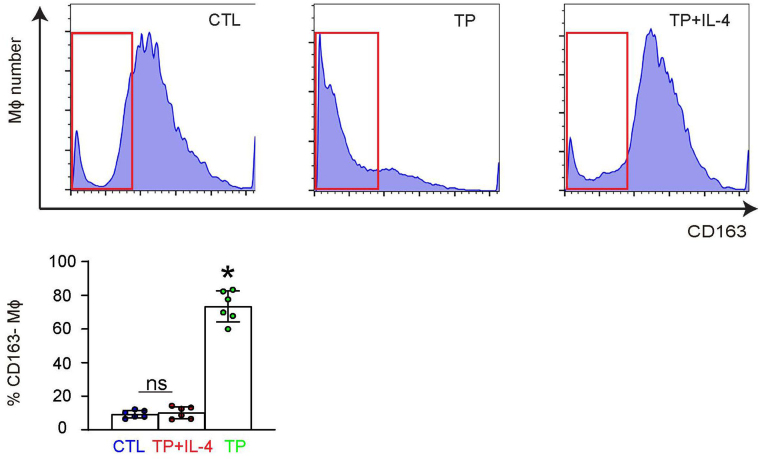
Significant anti-inflammatory TAMs are detected in triptolide-treated tumors that receive IL-4. The tumor was dissected out and dissociated into single cells at sacrifice. Afterwards the single cell preparation underwent FAC analysis, showing significant increases in pro-inflammatory fraction (CD163-) of the macrophages (F4/80+) in the tumors from Triptolide-treated mice, which were abolished by addition of IL-4 treatment.

**Figure 7:**
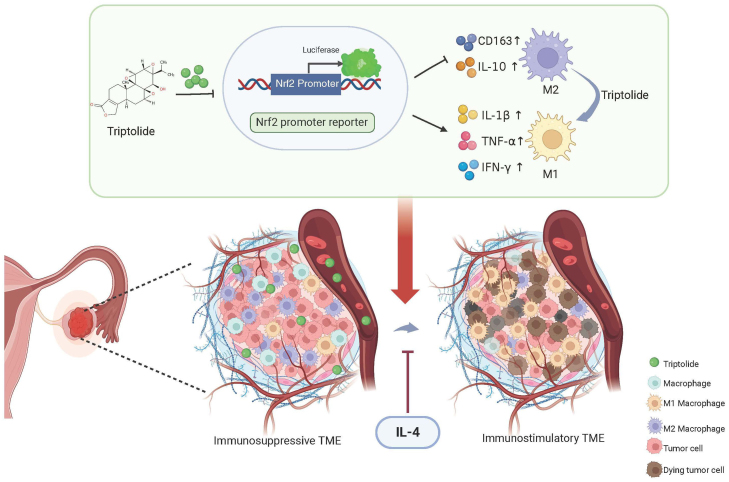
A proposed model: triptolide inhibits ovarian cancer growth and metastasis via reprogramming of tumor-associated macrophages.

In order to validate the effects of IL-4 on TAM polarization in the tumor, the tumor was dissected out and dissociated into single cells at sacrifice. Afterwards the single cell preparation underwent FAC analysis, showing significant increases in pro-inflammatory fraction of the macrophages (CD163-F4/80+) in the tumors from Triptolide-treated mice, which were abolished by addition of IL-4 treatment (Figure 6). Together, our study demonstrates that Triptolide Inhibits ovarian cancer growth and metastasis via anti-inflammatory reprogramming of tumor-associated macrophages (Figure 7).

## DISCUSSION

Our study highlights the significant role of the tumor microenvironment, particularly through TAMs, in Triptolide’s suppression of ovarian cancer 17. The suppressive effects of Triptolide on ovarian cancer, mediated through the tumor microenvironment, underscore the interplay between cancer cells and immune components 17. This compound modulates TAM polarization towards an anti-tumor M1 phenotype, a shift from their usual tumor-promoting M2 state 18. This reprogramming is pivotal, as M1 macrophages are known for their pro-inflammatory, tumoricidal activities, contrasting the M2 macrophages that support tumor growth and suppression of the immune response 18. Triptolide’s ability to induce such a shift highlights its potential as a therapeutic agent that leverages tumor immunology principles for cancer treatment 7, consistent with some previous reports 13–15.

On the other hand, it has also been shown that Nrf2 suppresses Il1b expression without affecting the majority of M1- or M2-induced genes 19. It is noteworthy that the M1/M2 dichotomy is an oversimplified model that does not fully capture the plasticity and functional complexity of macrophages, especially within the TME 20. A single gene or small panel (e.g., Il1b, Arg1) cannot conclusively define macrophage polarization as pro- or anti-inflammatory. Macrophage states exist on a functional spectrum influenced by metabolic, transcriptional, and environmental cues 21. Therefore, our interpretation does not contradict the findings of this previous report 19. Rather, tother with all these previous related studies 13–15, 19, we showed that under stress conditions such as cancer, Nrf2 may regulate a broader transcriptional and metabolic program far beyond Il1b suppression. Our results suggest that triptolide modulates macrophage phenotype at least in part by reducing Nrf2 expression and activity, leading to a shift away from TAM-associated features.

Triptolide’s impact on ovarian cancer progression, mediated through TAMs polarization from M2 to M1 phenotype, underscores the significance of epigenetic control in immune modulation 22. This transition is pivotal because M1 macrophages exhibit tumoricidal activities, including the production of pro-inflammatory cytokines, which contrast the tumor-promoting functions of M2 macrophages 22. The mechanism behind Triptolide’s action involves the inhibition of Nrf2 transcription, a master regulator of cellular defense mechanisms against oxidative stress 23. Given that the Nrf2 promoter is rich in CpG islands, which are key sites for epigenetic modifications such as methylation, Triptolide’s effect suggests a profound alteration in the epigenetic landscape of macrophages 24, 25. This alteration likely prevents the binding of transcription factors necessary for M2 polarization, thereby skewing the macrophage population towards an M1 phenotype 23. Such a shift not only enhances the immune system’s ability to combat cancer cells but also potentially reverses the immunosuppressive environment typically fostered by tumors 23. This dual action of Triptolide, affecting both cancer cells directly and the tumor microenvironment indirectly through epigenetic reprogramming of macrophages, highlights its therapeutic potential in targeting complex cancer mechanisms beyond cytotoxicity 23.

The enhanced effects of Triptolide on ovarian cancer cells, especially when co-cultured with macrophages, can be attributed to Triptolide’s ability to reprogram TAMs from an M2 to an M1 phenotype. M2 macrophages, known for promoting tumor growth and suppressing immune responses, are shifted towards the M1 phenotype, which exhibits tumoricidal activity and supports immune-mediated cancer cell destruction. This reprogramming thus not only targets cancer cells directly but also modifies the tumor microenvironment to further suppress cancer progression.

To summarize, our study’s findings underline the importance of the cancer microenvironment in therapy and open new avenues for using Triptolide in ovarian cancer treatment, emphasizing the need for further research into its epigenetic mechanisms.

## MATERIALS AND METHODS

### Ethics

Experimental and animal procedures in the current study have been approved by the research committee at Shanghai Municipal Hospital of Traditional Chinese Medicine (number: 2022SHL-KYYS-86) and were carried out in accordance with the approved guidelines.

Patients and the public were not (or will not be) involved.

### Cell culture and promoter assay

To isolate and culture human naïve macrophages from the bone marrow of healthy donors, begin by harvesting bone marrow and isolating mononuclear cells via density gradient centrifugation using Ficoll-Paque (specific gravity 1.077 g/mL). Next, plate these cells in Teflon-coated culture dishes with RPMI-1640 medium supplemented with 10% fetal bovine serum (FBS), 1% penicillin-streptomycin, and 20 ng/mL of macrophage colony-stimulating factor (M-CSF) to promote differentiation into macrophages. Maintain cultures at 37°C in a humidified atmosphere containing 5% CO2, refreshing the medium every 2–3 days. After 7–10 days, when cells have differentiated into naïve macrophages, they can be detached using gentle scraping. Cultured naïve macrophages are treated with interleukin-4 (IL-4; Sigma-Aldrich, St Louis, MO, USA) at a concentration of 20 ng/mL to induce the polarization of macrophages towards the M2 phenotype, which is associated with anti-inflammatory functions. Human ovarian cell lines A2780 and SKOV3 were both purchased from American Type Culture Collection (ATCC, Rockville, MD, USA). Both cell lines were maintained in Dulbecco’s Modified Eagle’s Medium (DMEM, Invitrogen, Carlsbad, CA, USA) supplied with 10% FBS. All cell lines were incubated in a humidified chamber with 5% CO ${}_{2}$ at 37°C. A2780 was derived from human ovarian endometrioid adenocarcinoma and are known to be sensitive to cisplatin. SKOV3 originate from human ovarian serous adenocarcinoma and are known for their aggressive growth and resistance to chemotherapeutic drugs like cisplatin. The human Nrf2 promoter from position − 1552 to +1091 was cloned into pcDNA3.1-CMV-luciferase vector supplied by Clontech (Mountain View, CA, USA) to generate a Nrf2 promoter reporter construct. Promoter assay was performed with different concentration of Triptolide to assess the direct effect of Triptolide on the transcription of Nrf2. Next, this construct was used to transfect macrophages. After transfection, treat macrophages with different concentration of Triptolide. Finally, measure luciferase activity using a luminometer. The luminescence signal reflects the transcriptional activity of the Nrf2 promoter under the tested conditions.

### Transfection and genetic modification of macrophages

To silence or overexpress Nrf2 expression, macrophages were transfected with specific small interfering RNA targeting human *NFE2L2* (si-Nrf2; sequence: 5’-GUAAGAAGCCAGAUGUUAAdTdT-3’) or a scrambled control siRNA (scr; sequence: 5’-UUCUCCGAACGUGUCACGUdTdT-3’), or an Nrf2 expression vector (Nrf2), was transfected into the cells. All transfections were performed using Lipofectamine 3000 (Thermo Fisher Scientific) according to the manufacturer’s instructions. The complete coding sequence (CDS) of human Nrf2 was amplified and cloned into a pcDNA3.1 expression vector to facilitate Nrf2 overexpression. The knockdown and overexpression efficiencies were validated by RT-qPCR and Western blot to ensure a significant reduction or increase in Nrf2 levels before the modified macrophages were utilized in co-culture experiments.

### Cellular viability assessment

A2780 and SKOV3 cells were plated at a density of 1 × 10 ${}^{4}$ cells per well in 96-well plates. These cells underwent treatment with Triptolide, ensuring that their density surpassed 60% at distinct time points: 0, 24, 48, and 72 hours. To measure cellular viability, we employed an CCK-8 assay kit, adhering to previously established protocols. Following a 2-hour incubation period with the CCK-8 solution, viability was quantitatively determined using a microplate reader to measure absorbance at 450 nm. This method allowed for an accurate evaluation of cell survival and proliferation in response to Triptolide treatment over the specified durations.

### Cell clock assay

To evaluate cell proliferation and apoptosis, we utilized two distinct assays. For cell proliferation, the Cell-Clock Cell Cycle Assay was employed, leveraging a redox-sensitive dye taken up by viable cells. This dye changes color according to the cell cycle phase: cells in G1 appear small, pale yellow, and circular; S phase cells are larger, light green, and resemble fibroblasts; G2 phase cells turn dark green and round; and cells in the M phase are characterized by an intense blue color and round shape. This colorimetric differentiation, provided by Biocolor LTD, Carrickfergus, UK, allows for the visual assessment of the cell cycle distribution. Specifically, the proportion of cells in the S phase relative to the total cell population was calculated as an indicator of cell proliferation in this study.

### Western blot analysis for Nrf2

To assess Nrf2 protein expression levels, Western blot analysis was performed on both control and triptolide-treated samples. Briefly, cells were lysed using RIPA buffer (50 mM Tris-HCl, 150 mM NaCl, 1% NP-40, 0.5% sodium deoxycholate, and 0.1% SDS) supplemented with protease and phosphatase inhibitors. The lysates were centrifuged at 12,000 × g for 15 minutes at 4°C, and the supernatants were collected for protein quantification using the BCA Protein Assay Kit (Thermo Fisher Scientific). Equal amounts of total protein (30–50 μ g per lane) were separated by 10% SDS-PAGE and subsequently transferred onto PVDF membranes (Millipore). Membranes were blocked with 5% non-fat milk in TBS-T (20 mM Tris-HCl, 150 mM NaCl, 0.1% Tween-20) for 1 hour at room temperature and incubated overnight at 4°C with a primary antibody against Nrf2 (1:1000 dilution; Cell Signaling Technology) and β -actin or GAPDH (1:5000 dilution; as a loading control). After washing, membranes were incubated with HRP-conjugated secondary antibodies (1:5000) for 1 hour at room temperature. Protein bands were visualized using an enhanced chemiluminescence (ECL) detection system (Bio-Rad) and quantified by densitometric analysis with ImageJ software. The relative expression of Nrf2 was normalized to the loading control and expressed as a fold-change compared with the control group.

### Transwell assays

To assess the migratory and invasive capabilities of SKOV3 cells, a Transwell assay was performed. In the migration assay, 5 × 10 ${}^{4}$ cells in 100 μ L of serum-free medium were placed into the upper compartment of the Transwell setup. To encourage cell migration, 800 μ L of medium supplemented with 10% fetal bovine serum (FBS) was introduced into the lower compartment. After allowing sufficient time for migration, the cells that traversed to the bottom side of the membrane were thoroughly rinsed with phosphate-buffered saline (PBS) three times, fixed with 4% paraformaldehyde for 30 minutes, and then stained with 1% crystal violet for visualization. The invasion assay was similarly conducted, with the additional step of coating the upper compartment with Matrigel to simulate the extracellular matrix barrier. The subsequent steps for fixation, staining, and analysis were identical to those of the migration assay. The results were examined and captured using a microscope at 100x magnification, providing visual evidence of cell migration and invasion capabilities.

### Mouse studies

Twelve-week-old male NOD/SCID mice, characterized by their immune deficiency and sourced from SLAC Laboratory Animal Co. Ltd in Shanghai, China, were selected as subjects for the experiment involving tumor cell implantation. A precise quantity of 1 × 10 ${}^{6}$ tumor cells was subcutaneously introduced into the lower left quadrant of the abdominal region of these mice (six mice per group). Triptolide was given to mice by i.p. injection at 55 μ g/kg at a frequency of twice a week. Control mice received saline injection. Besides the control group that received saline instead of TP, another TP-treated group received the administration of IL-4 (5 μ g per injection) to counter Triptolide’s influence on macrophage polarization. The progression and development of the tumor were meticulously monitored over a span of four weeks using calipers. Two dimensions are typically recorded: length (the longest dimension) and width (the dimension perpendicular to the length). These measurements are used to calculate the tumor volume, often using the formula for an ellipsoid: Volume = (length × width ${}^{\wedge }$ 2) × π /6. This formula approximates the volume of the tumor based on its external dimensions. Four weeks following the transplantation, the development of the tumors at the end point was measured by tumor weight.

### Flow cytometry

Flow cytometry was utilized for analyzing macrophage polarization. Tumors were harvested from mice, minced, and enzymatically dissociated into single-cell suspensions using 0.25 mg/mL trypsin (Thermo Fisher Scientific) for 35 minutes at 37°C with gentle agitation. Following digestion, samples were filtered through a 70- μ m cell strainer to remove undigested tissue and debris. Single-cell suspensions were washed, counted, and incubated with Fc block (anti-mouse CD16/CD32) to prevent nonspecific antibody binding. Cells were then stained with fluorophore-conjugated antibodies targeting F4/80 to identify macrophages. Macrophage subsets were further distinguished based on surface expression of CD86 (M1-like) and CD163 (M2-like). Appropriate isotype controls and fluorescence minus one (FMO) controls were included to define gating strategies and minimize background fluorescence. Data acquisition was performed on a FACSAria flow cytometer (BD Biosciences), and a minimum of 100,000 events per sample were recorded. Data were analyzed using FlowJo software (version 10; FlowJo LLC, Ashland, OR, USA). Gating was based on forward and side scatter to exclude debris and doublets, followed by identification of F4/80 ${}^{+}$ macrophages and assessment of CD86 ${}^{+}$ and CD163 ${}^{+}$ subpopulations to determine polarization status.

### ELISA

To collect proteins for ELISA analysis, cells were lysed using RIPA buffer. We employed a range of ELISA kits for analyzing various human cytokines and growth factors, following the manufacturer’s instructions. These included kits for human Interleukin-1 beta (IL-1 β ; KAC1211) from Invitrogen, human Tumor Necrosis Factor Alpha (TNF- α ) ab309419, Interferon Gamma (IFN- γ ) ab174443, CD163 ab274394, IL-10 ab185986 and Nrf-2 ab277397 from Abcam.

### TUNEL staining

TUNEL (terminal deoxynucleotidyl transferase dUTP nick end labeling) staining was performed using a TUNEL kit (Abcam, Shanghai, China; Ab66110).

### Statistical analysis

The statistical analyses were performed using GraphPad Prism (GraphPad Software, Inc., La Jolla, CA, USA). The data were presented as individual values with the mean and standard deviation. To compare more than two groups, one-way analysis of variance was used, followed by Tukey’s post hoc analysis. Significance was set at p < 0.05.

### Data availability statement

The datasets generated during and/or analyzed during the current study are available from the corresponding author on reasonable request.

## AUTHORS CONTRIBUTIONS

**Ziqi Chen:** Conceptualization, Investigation, Formal analysis, Validation, Writing - original draft, Writing - review & editing, Funding acquisition. **Jie Zhang:** Investigation, Writing-original draft, Funding acquisition. **Jianting Lao:** Investigation, Data curation. **Hong Yang:** Conceptualization, Project administration, Supervision, Funding acquisition. **Chaoqin Yu:**Project administration, Supervision. All authors read and approved the final manuscript.

## SUPPLEMENTAL MATERIAL

All supplemental data for this article are available online at http://www.cell-stress.com/researcharticles/2026a-chen-cell-stress/.

Click here for supplemental data file.

## CONFLICT OF INTEREST

The author has declared that no competing interests exist.

## ABBREVIATIONS

CCK-8 – cell counting kit-8

CD – cluster of differentiation

ELISA – enzyme-linked immunosorbent assay

FACS – fluorescence-activated cell sorting

FBS – fetal bovine serum

IC50 – half-maximal inhibitory concentration

IFN – interferon

IL – interleukin

M-CSF – macrophage colony-stimulating factor

NOD/SCID – non-obese diabetic/severe combined immunodeficiency

Nrf2 – nuclear factor erythroid 2-related factor 2

OC – ovarian cancer

PBS – phosphate-buffered saline

PVDF – polyvinylidene difluoride

RT-qPCR – reverse transcription quantitative polymerase chain reaction

SDS-PAGE – sodium dodecyl sulfate-polyacrylamide gel electrophoresis

siRNA – small interfering RNA

TAMs – tumor-associated macrophages

TNF – tumor necrosis factor

TP – triptolide

TUNEL – terminal deoxynucleotidyl transferase dUTP nick end labeling

WB – western blotting
