# Supplementary figures and images for: Triptolide inhibits ovarian cancer growth and metastasis via reprogramming of tumor-associated macrophages
Source: Cell Stress. 2026 Jul 7;10:53–63. doi: 10.15698/cst2026.07.319 (PMC13365739; doi:10.15698/cst2026.07.319)

Figure S1: Uncut gel for Figure 3B.

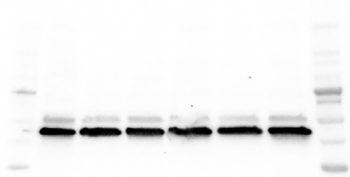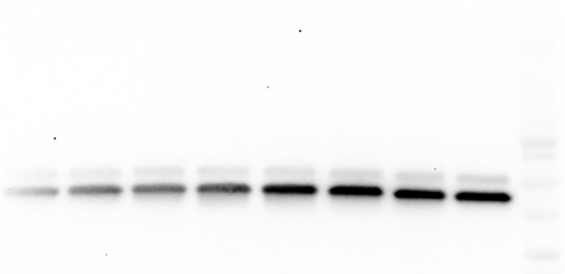

Figure 3B

Supplement: Supplementary file 1 [file ces-10-053-s01.pdf]
